# Craniofacial Changes Among Children and Adolescents Submitted to Growth Hormone Therapy: A Systematic Review

**DOI:** 10.1111/ocr.12937

**Published:** 2025-04-23

**Authors:** Raul Borges Nascimento, Suelly Maria Mendes Ribeiro, Nathalia Carolina Fernandes Fagundes, David Normando

**Affiliations:** ^1^ Faculty of Dentistry Federal University of Pará Belém Brazil; ^2^ Department of Orthodontics, Post‐Graduation Program in Dentistry Federal University of Pará Belém Brazil

**Keywords:** face, growth and development, growth hormone, orthodontics

## Abstract

The aim of this systematic review was to investigate the association between craniofacial changes and growth hormone (GH) therapy among children and adolescents with GH deficiency or idiopathic short stature (ISS). The PRISMA guideline was followed to carry out all stages of this review. An electronic search was conducted in seven databases, without year or language restrictions. The study selection was carried out in two stages by two calibrated examiners. Studies exploring craniofacial changes among children and adolescents with GH deficiency or ISS undergoing GH therapy were included. After data extraction, the risk of bias was assessed using the Joanna Briggs Institute (JBI) Critical Appraisal tool and the RoB 2.0 Checklist. The certainty of the evidence was assessed using the GRADE tool. Among the 4494 identified citations, seven studies met the eligibility criteria. These studies evaluated the impact of GHs on cephalometric measurements and dental age. No differences between immediate and delayed treatments were found in maxillary and mandibular dimensions. All selected articles showed a low risk of bias. A low certainty of evidence was observed for all outcomes assessed. GH therapy appears to result in a small increase in mandibular dimensions, although without clinical significance characteristic of adverse effects. Clinical trials and long‐term follow‐up studies of these patients are needed to develop accurate recommendations regarding the effects of GHs in the craniofacial region. Growth hormone may result in a slight increase in mandibular and maxillary dimensions, without significant adverse effects to general health. Controlled intervention studies with long‐term follow‐up are needed to establish more precise recommendations.

**Trial Registration:** PROSPERO database (https://www.crd.york.ac.uk/prospero/): CRD42024511329

## 1 Introduction

The regulation of postnatal craniofacial growth is determined by interactions between genes, hormones and nutrients, with local control of growth and bone remodelling being influenced by tissues adjacent to skeletal structures [1]. In childhood and adulthood, growth delay can cause functional and social impairment to the individual, such as orthopaedic problems, difficulties in social interaction and challenges in the job market [2]. As possible consequences for the craniofacial complex, a higher frequency of mandibular retrognathia, a more open gonial angle and an increase in lower facial height have been reported, characteristics frequently observed in children with a history of a decrease in the normal growth curve [3].

One of the alternatives to modulate the growth rate in children is growth hormone (GH) therapy. GH is produced and secreted by the pituitary gland and helps modulate endochondral bone growth [4]. Currently, the most widely accepted theory is that GH can stimulate bone growth through the proliferation of chondrocytes and osteoblasts, promoting an increase in the bone deposition and remodelling rate [5]. The use of GH therapy is strongly recommended for children and adolescents with severe and permanent GH deficiency, with an initial GH dosage of 0.16–0.24 mg/kg/week and average duration varying from 1 to 7 years [6]. A combination of features, including growth phase at the beginning of therapy, the growth velocity during GH therapy and the risk of potential adverse effects of GH therapy (e.g., development of intracranial hypertension, slipped capital femoral epiphysis and scoliosis progression) may determine the specific dosage and duration of the therapy [6, 7].

Additionally, a weaker recommendation supports the use of GH therapy in cases of idiopathic short stature (ISS) [6]. In this scenario, the dosage strategy and treatment duration must be established based on a shared decision‐making process assessing the physical and psychological burdens to the patient, as well as the risks and benefits of the treatment [7, 8].

The relationship between GH and craniofacial growth has been studied. It has been suggested that GH administration in children may accelerate craniofacial growth, mainly affecting posterior facial height, which may lead to the correction of potential problems [4]. A previous systematic review reported that GH therapy may result in increased maxillary [9], mandibular and cranial base length. However, questions associated with the influence of the administered GH dosage, the association between the type of growth deficiency and craniofacial changes, including the occurrence of adverse effects on craniofacial growth, remain unclear and still need to be summarised. This review aimed to investigate the association between craniofacial changes and GH therapy among children and adolescents with GH deficiency or ISS. As a secondary aim, the influence of GH dosage and duration of therapy were also explored.

## 2 Materials and Methods

### 2.1 Protocol and Registration

The protocol for this review was registered in the PROSPERO database, with registration code CRD42024511329. This review followed the Preferred Reporting Items for Systematic Reviews and Meta‐Analysis (PRISMA) guidelines [10].

### 2.2 Eligibility Criteria

The studies were selected according to established criteria and based on the acronym PECO. The population (P) of choice was children and adolescents with growth hormone deficiency (GHD) or ISS; regarding exposure (E), patients undergoing GH therapy were included; the comparison (C) was established with patients not treated with GH; and the outcome (O) was changes in craniofacial growth after treatment. The study design included case–control and cohort studies and clinical trials focusing on the evaluation of craniofacial growth in children undergoing GH therapy. The exclusion criteria included studies with patients who had craniofacial syndromes, used orthodontic appliances before the intervention, review articles, opinion articles, technical articles, guidelines, case reports, animal and in vitro studies.

### 2.3 Search Strategy and Study Selection

The search strategy was developed using MeSH terms and keywords related to the PECO acronym. The searches were conducted in November 2024, by searching five electronic databases: PubMed, Cochrane, Web of Science, LILACS, Embase and Medline via Ovid using the Boolean operators ‘OR’ and ‘AND’ to adapt the search strategy to each database (Appendix S1, Table S1). The ProQuest Dissertations and Theses database was used as a source for searching the grey literature. After the searches, all citations were exported to a systematic review management software (Rayyan, Qatar Computing Research Institute, Doha, Qatar) [11].

Two reviewers (R.B.N. and S.M.M.R.) independently selected articles included in both phases. In the first phase, duplicate articles were removed, and the remaining articles were screened based on their titles and abstracts, following the eligibility criteria. In the subsequent phase, the full texts of potentially relevant studies were thoroughly analysed, assessed and selected according to the same eligibility criteria adopted in the first phase. Subsequently, a cross‐comparison of all identified information was performed. In case of discrepancies during the selection, two other authors (N.C.F.F. and D.N.) were consulted.

### 2.4 Data Extraction

Data extraction from the studies computed information on authorship, year of publication, study design, sample characteristics (sample size and age), bone growth assessment method, GH application protocol, follow‐up period and main results. Two calibrated examiners (R.B.N. and S.M.M.R.) collected data and later shared it among the other authors (N.C.F.F. and D.N.) to check for discrepancies.

### 2.5 Risk of Bias

The risk of bias was assessed according to the methodological characteristics of the studies. For cohort studies, the Joanna Briggs Institute Critical Appraisal Tool for Cohort and Cross‐sectional Studies were used [12]. This tool presents a specific checklist with 10 questions related to the methodological quality of the studies evaluated, each of which was judged as ‘yes’, ‘no’, ‘unclear’ and ‘not applicable’. The articles included were judged as high risk (yes score ≥ 49%), moderate risk (yes score = 50%–69%) and low risk (yes score ≥ 70%) [13]. The Cochrane risk‐of‐bias tool for randomised trials—ROB 2.0 was applied for the randomised controlled trials (RCT) included in this review [14]. The evaluation was performed by two reviewers (R.B.N. and S.M.M.R.) and disagreements were resolved by two reviewers (N.C.F.F. and D.N.).

### 2.6 Certainty of Evidence

The GRADE (Grading of Recommendations, Assessment, Development and Evaluations Pro) software (GRADEpro, available at https://www.gradepro.org/) was used to assess the certainty of the evidence. The domains were assessed for three outcomes: changes in facial height, mandibular dimensions and maxillary dimensions. This tool classifies each of the five domains assessed—study design, risk of bias, inconsistency, indirect evidence and imprecision of articles—as ‘not serious’, ‘serious’ or ‘very serious’. From this, the final quality of the evidence is categorised into four levels: very low, low, moderate and high [15].

## 3 Results

### 3.1 Study Selection

**FIGURE 1:**
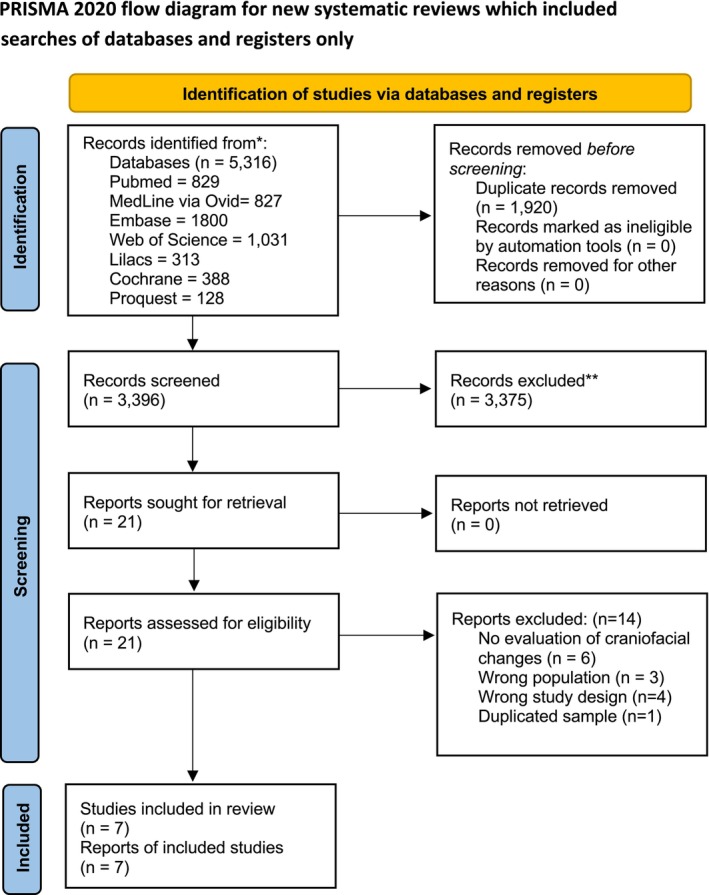
PRISMA flow chart. *Consider, if feasible to do so, reporting the number of records identified from each database or register searched (rather than the total number across all databases/registers). **If automation tools were used, indicate how many records were excluded by a human and how many were excluded by automation tools. *Source:* Page et al. [10].

Database searches identified 5316 citations: Cochrane Library (*n* = 388), Web of Science (*n* = 1031), PubMed (*n* = 829), ProQuest (*n* = 128), Medline via Ovid (*n* = 827), Lilacs (*n* = 313) and Embase (*n* = 1800). After removing duplicate citations (*n* = 1920), 3396 potentially eligible studies remained. After analysing the titles and abstracts, 3375 articles were excluded, and 28 publications were selected for full‐text evaluation. Of these, 14 were removed; reasons for exclusion included lack of assessment of craniofacial growth, evaluation of patients with systemic syndromes or conditions, duplicate samples, journal editorials and cross‐sectional studies, resulting in the selection of seven studies for qualitative synthesis. The procedure for identifying and selecting studies is detailed in Figure 1.

### 3.2 Study Characteristics

Among the seven included studies, four were cohort studies [3, 16, 17, 18], two were cross‐sectional studies [19, 20] and one was a randomised clinical trial [21]. Regarding treatment time, two studies divided patients into a short‐term treatment group (< 2 years), a long‐term treatment group (> 2 years) and a control group [17, 19]. Another study categorised the samples into two groups: patients who received GH doses for less than 1 year and those who received doses for more than 1 year [20]. The remaining studies performed supplementation for 2 [16, 21], 3 [3] and 4 years [18].

Considering the type of growth alteration, studies with children with GHD [3, 16, 17, 18, 19, 21] and ISS [16] were included. According to the time patients received GH therapy, in the study conducted by de Faria et al. [3], GH administration was evaluated prospectively, with one group starting treatment during the study, whereas another group was already on GH treatment before the start of the research. Regarding the administration of GH doses, six studies applied doses of 0.1–0.3 mg/kg/week. In only one study, supplementation was carried out with doses of 0.2–0.4 mg/kg/week [18].

Regarding the duration of follow‐up, five studies followed patients for 2 years [16, 17, 19, 20, 21], and one study carried out follow‐up for 3 years [3]. Regarding the craniofacial analysis methods, five studies evaluated cephalometric aspects [3, 16, 17, 18, 21], and two evaluated dental age [19, 20]. A summary of the extracted data is available in Table 1.

**TABLE 1:** Data extracted from the included studies.

Author/Year/Country/Study design	Sample size	Age^a^ (years)	Rationale for GH therapy	Craniofacial features evaluated	Main results
Van Erum et al./1997/Belgium/RCT	*N* = 21 GH 0.2 mg/kg = 9 GH 0.3 mg/kg = 8 Control = 4	2–8	Small for gestational age	Cephalometric features (NS, S‐Ba, N‐Ba, N‐ANS, S‐PNS, ANS‐Me, N‐Me, S‐GO, ANS‐PNS, Art‐Go, GO‐Pog, Art‐Pog; NS.Art, Art.Go.Me, SN.GO.GN, SNA, SNB, ANB, SN Art.Go)	GH therapy at doses of 0.2 and 0.3 mg/kg/day promoted growth‐accelerating effects in the sample when compared to control group, especially in total mandibular length (0.2 mg = 86.3 ± 5.1 mm, 0.3 mg = 87.8 ± 5.4 mm, control = 84.0 ± 8 mm), total anterior facial height (0.2 mg = 104.2 ± 9.0, 0.3 mg = 103.5 ± 4.2 mm, control = 95.4 ± 2.3 mm) and cranial base length (0.2 mg = 95.4 ± 3.4 mm, 0.3 mg = 95.5 ± 2.4 mm, control = 91.2 ± 3.6 mm), when compared to the control group. A higher increasement in the posterior facial height was observed for the 0.3 mg group (62.8 ± 5.2 mm) when compared to the 0.2 mg (59.8 ± 5.2 mm) and control group (55.1 ± 10 mm) *p* < 0.05
Cantu et al./1997/USA/CS	*N* = 40 GH 0.2 mg/kg (for less than 2 years) = 13 GH 0.2 mg/kg (for more than 2 years) = 13 Control = 14	5–18	GH deficiency	Dental age, cephalometric features (SN, S‐Ba, PNS‐ANS Co‐Pg, Co‐Go, Go‐Pg, N‐Me, S‐Go)	GH therapy was effective in restoring growth in all cases, without adverse effects. GH therapy > 2 years was more effective in stimulating delayed craniofacial growth compared to GH treatment < 2 years for anterior facial height, posterior facial height and posterior cranial base length. *p* < 0.05
Funatsu et al./2006 Japan/CH	*N* = 57 GH 0.2 mg/kg (≤ 2 years): 17 GH 0.2 mg/kg (> 2 years): 23 Control: 17	10–12	GH deficiency	Cephalometric features (NS, N‐Me, N‐ANS, ANS‐Me, A‐Ptm, Gn‐Cd, Pog‐Go, Cd‐Go)	The standard deviation scores for upper facial height (Control 49.4 ± 3.0, GH < 2 years—52.9 ± 6.3, GH > 2 years—54.6 ± 5.8); maxillary length (Control 44.8 ± 2.3, GH < 2 years—45.4 ± 3.3, GH > 2 years—47.2 ± 2.9) and mandibular ramus height (Control 49.0 ± 3.7, GH < 2 years—52.2 ± 3.3, GH > 2 years—54.7 ± 5.4) were significantly increased when compared with the control group. *p* < 0.05
Kawala et al./2007/Poland/CS	*N* = 25 GH 0.2 mg/kg (< 1 year) = 6 GH 0.2 mg/kg (> 1 year) = 19	7–14	GH deficiency	Dental age, cephalometric features (SNA, SNB, ANB and goniac angle)	Forward mandibular growth (SNB) was significant (0.6° greater, on average) in the group with longer treatment. No adverse effects were observed in both groups
Kjellberg and Wikland/UK/2007/CH	*N* = 176 GH 0.2 mg/kg = 31 GH 0.4 mg/kg = 15 Control = 109	11.2 ± 1.7	Idiopathic short stature and GH deficiency	Cephalometric features (S–N–SS, S–N–SM, S–N–PG, N–SS–PG, GN–TGO–AR, N–S–BA, N–S–AR, S–N, S–BA, SP′–GN, TGO‐AR, GN‐TGO, AR–GN, N‐GN, TGO′–TGO, SP′–GN, N–SP′, PM–ANS)	Significantly accelerated growth was observed for patients treated with GH in the mandibular length (GN‐TG0: MD = 0.8 mm and AR–GN: MD = 1.5 mm) and total and lower anterior facial height (N–GN: MD = 1.4 mm and SP′–GN: MD = 1.4 mm) when compared to the controls. No differences were observed in relation to the different GH therapy dosage
de Faria et al./2009 Brazil/CH	*N* = 20 GH 0.6 mg/kg (3 years) = 6 GH 0.6 mg/kg (6.5 years) = 14	2–11	GH deficiency	Cephalometric features (SN, S‐Ar Co‐Gn, Co‐A, N‐Me, NS‐Me)	In the GH 0.6 mg/kg group (3 years), significant growth was observed in the posterior base of the skull (MD = 0.9), mandible (MD = 3.1) and lower anterior facial height (MD = 0.5) when compared to the measurements obtained at the beginning and after 3 years of study. *p* < 0.05
Choi et al., 2017/korea/CH	*N* = 36 GH deficiency = 18 Idiopathic short stature = 18 Control = 18	11.3 ± 1.8	GH deficiency and idiopathic short stature	Cephalometric features (NS, S‐Ba, ANS‐PNS, Ar‐Go, Go‐Gn, Ar‐Gn, ANS‐Me, N‐Me, S‐Go; Angular: NS‐Ar, SN‐ArGo, Ar‐GoMe, SNA, SNB, ANB)	Boys with GH deficiency had greater convexity of the maxillomandibular relationship (ANB: 3.20 ± 1.55) than controls (ANB: 1.7 ± 0.7). Girls with idiopathic short stature (109.3 ± 3.6) had a significantly greater mandibular growth than those with GH deficiency (104.88 ± 2.08). The anterior facial height in girls with idiopathic short stature (128.19 ± 5.95) was greater than controls (119.5 ± 6.2) *p* < 0.01

Abbreviations: CH, cohort; CS, cross‐sectional; GH, growth hormone; MD, mean difference; RCT, randomised controlled trial.

^a^ Depending on the data reported by the included papers, age was reported as range or mean ± standard deviation.

### 3.3 Individual Results of Included Studies

Among the included studies, children and adolescents under GH therapy presented a more pronounced growth in facial height, mandibular length and mandibular ramus height and maxillary length [3, 16, 17, 18, 19, 20, 21].

The total anterior and posterior facial height, total anterior facial height and anterior lower facial height were explored across studies. Anterior facial height was reported to be more pronounced among children undergoing GH therapy in one study that explored GH dosages between 0.2 and 0.3 mg/kg/day among children and adolescents from 2 to 8 years, in which the mean difference varied from 7 to 8 mm in one study [21], and 0.5 *z*‐score (i.e., the number of standard deviations below or above the reference mean or median value for an anthropometric measurement) in another study when compared to untreated control groups. A slightly more pronounced increase in total anterior facial height was observed among patients being managed with a slightly higher GH dosage (i.e., 0.2 mg/kg/day when compared to 0.3 mg/kg/day) [21]. The GH therapy group, when compared to untreated controls, also showed an upper facial height of 5 mm higher in one study [17], and a posterior facial height of 7 mm higher in another study [21].

Two studies that only investigated before and after results after different dosages of GH therapy also reported a more pronounced lower anterior facial height varying from 0.2 to 1.1 mm among children from a wide age range (1.5–18 years) [3, 18].

Regarding the reported more pronounced growth on mandibular length and mandibular ramus height of children under GH therapy, one study investigated and observed a 3 mm higher overall mandibular length when compared to an untreated group [21], whereas one study investigated and reported a mandibular ramus height 4 mm higher when compared to the untreated group [17]. Two studies that only investigated before and after results after different dosages of GH therapy also reported a 0.7–1.5 mm more pronounced overall mandibular length [3, 18].

Regarding the maxillary and cranial base differences, one study investigated and observed that maxillary height was 2 mm higher when compared to an untreated control group [17], whereas one study observed an increase of 1 mm in the posterior cranial base after GH therapy [3].

Regarding the differences between sex, four studies explored possible craniofacial growth differences between boys and girls under GH therapy [3, 16, 17, 19]. One study reported a greater convexity of the maxillomandibular relationship among boys with GHD under GH therapy (ANB: 3.20° ± 1.55° [*p* < 0.01]) than that of the control group (ANB: 1.77° ± 0.75° [*p* < 0.01]) [16].

Regarding the differences in facial growth according to the origin of growth deficiency, girls with ISS (109.38 ± 3.62 mm [*p* < 0.01]) presented a significantly greater amount of mandibular growth than those with GHD (104.88 ± 2.08 mm [*p* < 0.01]) during GH treatment, when compared to those considered healthy (control group). Furthermore, the anterior facial height in girls with ISS (128.19 ± 5.95 mm [*p* < 0.01]) was greater than in the reference group (119.56 ± 6.26 mm [*p* < 0.01]), 2 years after the start of GH treatment [16]. In another study involving patients with GHD, significantly accelerated growth was observed for patients treated with GH in the length of the mandibular body (GN‐TG0: MD = 0.86 mm and AR–GN: MD = 1.58 mm) and total and lower height of the anterior face (N–GN: MD = 1.45 mm and SP′–GN: MD = 1.43) when compared to the control group. No significant differences were observed for any of the variables concerning the different doses and regarding the presence of GHD or ISS [18].

Patients undergoing therapy with doses of 0.6 mg/kg for up to 3 years presented significant growth in the posterior base of the skull (MD = 0.98), mandible (MD = 3.14) and lower third of the face (MD = 0.56) when compared to the measurements obtained at the beginning and after 3 years of study [3]. During a treatment period longer than 2 years, patients exhibited a more significant increase in maxillomandibular dimensions (52.9 ± 6.3 mm [*p* < 0.05]), in contrast to those who were treated for less than 2 years (54.6 ± 5.8 mm [*p* < 0.05]) [17, 18].

Regarding the development of adverse effects to general health or pertinent to craniofacial growth, none of the included studies reported adverse effects to general health or craniofacial growth in either children or adolescents with GHD or ISS [3, 16, 17, 18, 19, 21].

### 3.4 Risk of Bias

**FIGURE 2:**
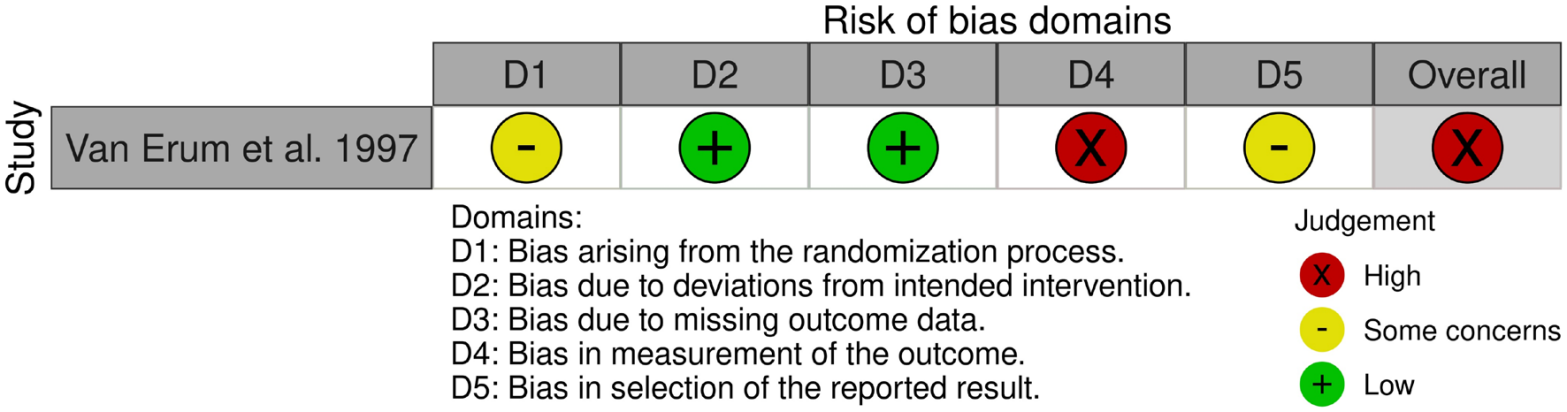
Risk of bias analysis for the randomised controlled studies included in the sample (*n* = 2).

Six included studies demonstrated a low risk of bias and one study presented a high risk of bias. The latter was categorised as high risk due to some concerns in the randomisation process, some concerns in the selection of the reported results and bias in the measurement of the outcome, in which the control group presented a different measurement technique than used for the treatment groups and there was an absence of information on a pre‐specified analysis plan of the results [21]. In addition, even with a low risk of bias, some minor methodological problems were observed, such as the presence of groups not collected from the same population [16], and in addition, a limited number of participants in the samples examined was also observed. The results of the risk of bias assessment are available in Figure 2 and Tables 2 and 3.

**TABLE 2:** Risk of bias assessment for cross‐sectional studies (*n* = 2).

	Cantu et al. (1997) [19]	Kawala et al. (2007) [20]
Were the sample inclusion criteria clearly defined?	Y	Y
Were the study subjects and environment described in detail?	Y	Y
Was exposure measured validly and reliably?	Y	Y
Were objective and standardised criteria used to measure the condition?	Y	Y
Have confounding factors been identified?	N	N
Have strategies for dealing with confounding factors been stated?	NA	NA
Were the results measured validly and reliably?	Y	Y
Was appropriate statistical analysis used?	Y	Y
Final RoB	Low	Low

Abbreviations: *N*, no; NA, question not applicable; RoB, risk of bias; U, unclear; Y, yes.

**TABLE 3:** Risk of bias analysis for the cohort studies (*n* = 4).

	Choi et al. (2017) [16]	de Faria et al. (2009) [3]	Kjellberg and Wikland (2007) [18]	Funatsu et al. (2006) [17]
Were the two groups similar and recruited from the same population?	N	U	Y	Y
Were the exposures measured similarly to assign people to both exposed and unexposed groups?	Y	Y	Y	Y
Was the exposure measured in a valid and reliable way?	Y	Y	Y	Y
Were confounding factors identified?	N	N	N	N
Were strategies to deal with confounding factors stated?	NA	NA	NA	NA
Were the groups/participants free of the outcome at the start of the study (or at the moment of exposure)?	Y	Y	Y	Y
Were the outcomes measured in a valid and reliable way?	Y	Y	Y	Y
Was the follow‐up time reported and sufficient to be long enough for outcomes to occur?	Y	Y	Y	Y
Was follow‐up complete, and if not, were the reasons to loss to follow up described and explored?	Y	Y	Y	Y
Were strategies to address incomplete follow‐up utilised?	NA	NA	NA	NA
Was appropriate statistical analysis used?	Y	Y	Y	Y
Final RoB	Low	Low	Low	Low

Abbreviations: *N*, no; NA, question not applicable; RoB, risk of bias; U, unclear; Y, yes.

### 3.5 Certainty of the Evidence

The certainty of the evidence was analysed based on three outcomes: changes in facial height [16, 17, 19], mandibular dimensions [3, 16, 18, 19] and maxillary dimensions [20]. The certainty of the evidence observed was very low for the three outcomes. The main reason for this result was the studies' lack of exploration of potential confounding factors. The quality of the articles was assessed by the GRADE system described in Table 4.

**TABLE 4:** Certainty of evidence assessed by GRADE tool.

Certainty assessment							Summary of findings		
Participants (studies) follow‐up	Risk of bias	Inconsistency	Indirectness	Imprecision	Publication bias	Overall certainty of evidence	Study event rates (%)		Impact
							With control	With GH	
**Changes on facial height (follow‐up: range 1–6 years)**									
306 (4 non‐randomised studies)	Very serious^a^	Not serious	Not serious	Not serious	All plausible residual confounding would suggest spurious effect, whereas no effect was observed	⨁◯◯◯ Very low	Four cohort studies evaluated short and long‐term changes in total anterior facial height, lower anterior facial height, upper anterior facial height and posterior facial height after GH therapy among children with idiopathic short stature and growth hormone deficiency. Total facial height and lower facial height seem to be increased in a long‐term evaluation (6 years after treatment), whereas controversies remain regarding the clinically significant short‐term effect (1–3 years) on the other facial height variables		
**Changes on mandible dimensions (follow‐up: range 1–6 years)**									
331 (5 non‐randomised studies)	Very serious^a^	Serious^b^	Not serious	Not serious	All plausible residual confounding would suggest spurious effect, whereas no effect was observed	⨁◯◯◯ Very low	Mandibular corpus length and mandibular plane seem to be increased in a short and long‐term evaluation (1 and 6 years after treatment), whereas a slightly increased but not clinically significant changes were observed on mandibular ramus height after GH therapy		
**Changes on maxillary dimensions**									
57 (1 non‐randomised study)	Very serious^a^	Not serious	Not serious	Not serious	All plausible residual confounding would suggest spurious effect, whereas no effect was observed	⨁◯◯◯ Very low	One cohort study evaluated changes in the maxillary length (A‐Ptm) after GH treatment among children with idiopathic short stature and growth hormone deficiency. A slightly increased but not clinically significant changes were observed after treatment without a follow‐up period		
**Changes on arch dimensions**									
31 (1 non‐randomised study)	Very serious^a^	Not serious	Not serious	Not serious	All plausible residual confounding would suggest spurious effect, whereas no effect was observed	⨁◯◯◯ Very low	No changes were observed on arch dimensions after treatment without a follow‐up period		

Abbreviation: CI, confidence interval.

^a^ Some concerns in randomisation process, selection of reported results and bias in measurement of the outcome of one of the studies.

^b^ Large variation in 95% confidence intervals was observed across studies.

## 4 Discussion

This review investigated the association between craniofacial changes and GH therapy in children and adolescents. It has been observed that GH therapy might result in increased maxillary and mandibular length, as well as total anterior and posterior facial height. Furthermore, the duration of treatment appears to be associated with the observed effects: patients undergoing GH therapy for a period longer than 2 years demonstrated an average increase of 2 mm more in mandibular dimensions compared with those treated for a period shorter than 2 years [17, 18].

In patients undergoing therapy with doses of 0.6 mg/kg/week for up to 3 years, significant growth was observed in the posterior base of the skull (MD = 0.98), mandible (MD = 3.14) and lower third of the face (MD = 0.56) when compared to measurements obtained at the beginning and after 3 years of study [3]. Regarding the effect of therapy with high doses of GH (0.3 mg/kg/day), faster craniofacial growth was observed, especially total mandibular length (GH = 87.8 ± 5.4 mm, control = 84.0 ± 8.0 mm), total anterior facial height (GH = 103.5 ± 4.2 mm, control = 95.4 ± 2.3 mm) and cranial base length (GH = 95.5 ± 2.4 mm, control = 91.2 ± 3.6 mm), when compared to the control group [21]. These results suggest that higher doses may stimulate bone growth in specific areas of the face and skull, which may be particularly relevant for patients with GH deficiency or conditions that affect craniofacial growth. Jaw and facial bone growth can positively impact facial aesthetics, masticatory function and the patient's overall health.

This review suggests that the effects of GH therapy on the craniofacial complex are more significant in the mandible, specifically in the mandibular body (mean difference of 1.58 mm concerning the control group) [16, 17, 18]. One hypothesis to justify this finding would be the presence of endochondral bone formation centres in the mandible—where a cartilage matrix is replaced by bone through chondrocyte maturation [22]. Furthermore, GH can stimulate bone growth by stimulating chondrocyte cell proliferation, resulting in increased production of cartilage tissue [23]. The higher the doses of GH to achieve a growth‐promoting effect, the greater the risk of triggering acromegalic effects [24]. However, in the study by van Erum et al. [21], included in this review, no adverse effects of GH supplementation were observed, indicating safety in use for more than 2 years.

Regarding the effect of duration of supplementation and craniofacial consequences, it was observed that patients who received GH doses for less than 1 year of therapy had lower craniofacial growth when compared with those who received it for more than 1 year, with anterior mandibular growth (SNB angle) being significantly greater (0.65°, on average) in the group with more extended treatment [20]. The difference observed in craniofacial growth, especially in the SNB angle, suggests that prolonged treatment with GH is more effective in promoting bone growth in the mandibular region. This may be crucial for patients with growth deficiency that affects the position and development of the mandible.

Considering the influence of GH therapy on different types of growth disorders, more specifically GHD and ISS, controversial results were observed among the included studies. Although one study did not identify a significant difference in hormonal influence on the craniofacial complex between patients with both disorders [18], two studies that included only female patients observed a greater tendency for mandibular growth in patients with ISS than in those with GHD, with an average difference of 4.5 mm [16]. These results suggest that the growth of the interstitial cartilage of the condyles is more influenced by GH in girls with ISS than in girls with GHD, which may indicate that, in these patients, the condylar cartilage has a greater sensitivity or an adaptive response that allows for more robust growth. Furthermore, when anterior facial height was taken into account, patients with ISS showed a growth of 8.63 mm greater compared to the control group [16].

It was also noted that GH treatment among children and adolescents born small for gestational age may result in compensation of craniofacial growth. This effect is especially evident in regions with interstitial cartilage growth, such as the mandibular body (total mandibular length [GH = 87.8 ± 5.4 mm, control = 84.0 ± 8.0 mm], total anterior facial height [GH = 103.5 ± 4.2 mm, control = 95.4 ± 2.3 mm] and cranial base length [GH = 95.5 ± 2.4 mm, control = 91.2 ± 3.6 mm], when compared to the control group) [21]. These effects indicate that GH treatment in children born small for gestational age can result in significant changes in craniofacial structure, requiring careful clinical monitoring to ensure that growth is harmonious and that functional or aesthetic problems are identified and corrected early.

Although this review may highlight somewhat positive results of GH therapy on craniofacial growth, the magnitude of this increase presented a large variability across included studies and presented a very low certainty of evidence. Thus, it is not yet possible to draw conclusions regarding a possible indication of GH therapy to address craniofacial growth deficiencies directly.

This review has important limitations. In addition to the fact that the included articles had a limited sample size, reducing the confidence in extrapolating the results to the general population, most studies presented a wide range in the age of the patients. Furthermore, the studies did not show an effect of GH therapy for a longer period (4 years or more).

This study also had limitations related to the risk of bias. One selected publication presented a high risk of bias, in which one study presented some concerns regarding the randomisation process and outcome measurement, presenting a variation of 90% in the confidence intervals of the reported results [21]. These flaws contributed to a very low certainty of evidence for the three outcomes assessed: changes in facial heights, changes in mandibular dimensions and changes in maxillary dimensions (Table 3). These limitations may indicate unclear or distorted conclusions about the effectiveness of the intervention, compromising the validity and reliability of the study results, making it important to interpret the findings with caution and consider the need for additional studies with more rigorous methodologies.

Also, this review highlights a scarcity of well‐conducted studies on the topic, which limited further generalisations of the presented results. Ideally, a methodologically sound study designed to investigate craniofacial growth differences between children under GH therapy would include a group of children exposed to this therapy compared to an untreated group of children from the same source or matched samples with similar demographic and craniofacial features. From the small sample size and limitations reported from the included studies in the present review, we may list a few methodological barriers that must be overcome in future investigations: difficulties in identifying and recruiting an untreated control group, difficulties in identifying and recruiting children with a homogenous craniofacial pattern, difficulties in assessing long‐term craniofacial growth.

Future studies are needed and should include validated assessment methods of proven accuracy, such as longitudinal studies to analyse the possible consequences of hormone supplementation for a longer period (6 years or more), as well as studies including a larger number of individuals. It is also necessary to conduct research with patients undergoing orthodontic treatment, directly demonstrating the influence of GH therapy on the treatment of these patients, directing the orthodontist's actions.

## 5 Conclusions

Growth hormone therapy appears to slightly increase mandibular and maxillary dimensions, although without clinically significant adverse effects to general health or craniofacial development. Controlled intervention studies with long‐term follow‐up are needed to establish more precise recommendations regarding the impact of GHs on the craniofacial region.

## Author Contributions

R.B.N. contributed to study design, data collection, data analysis and manuscript drafting. S.M.M.R. contributed to data collection and data analysis. N.C.F.F. contributed to study design, data analysis, manuscript revision and proofreading. D.N. contributed to study conceptualisation, study design, manuscript revision and proofreading. All authors read and approved the final manuscript.

## Ethics Statement

The authors have nothing to report.

## Conflicts of Interest

The authors declare no conflicts of interest.

## Supporting information


Appendix S1

Table S1

## Data Availability

The data supporting this study's findings are available in the main article and in the Supporting Information.
