# Supplementary material for: Craniofacial Changes Among Children and Adolescents Submitted to Growth Hormone Therapy: A Systematic Review
Source: Orthod Craniofac Res. 2025 Apr 23;28(5):772–82. doi: 10.1111/ocr.12937 (PMC12418064; doi:10.1111/ocr.12937)
Supplement: Supplementary file 2 — Table S1 [file OCR-28-772-s001.docx]

## Supplemental Files

Supplementary Table 1: Terms used on database search.

| Database | Search format |
| --- | --- |
| PUBMED  (n=829) | ("child*"[All Fields] OR "pediatric*"[All Fields] OR "paediatric*"[All Fields] OR ("elementary"[All Fields] AND ("schooler"[All Fields] OR "schoolers"[All Fields])) OR "schoolchild*"[All Fields] OR "boy"[All Fields] OR "girl*"[All Fields] OR (("middle"[All Fields] OR "middles"[All Fields]) AND ("schooler"[All Fields] OR "schoolers"[All Fields])) OR "pubescen*"[All Fields] OR "juvenile*"[All Fields] OR "teen*"[All Fields] OR "youth*"[All Fields] OR "adolesc*"[All Fields] OR "pre pubesc*"[All Fields] OR "prepubesc*"[All Fields]) AND ("craniofacial*"[All Fields] OR "face*"[All Fields] OR ("maxilla"[MeSH Terms] OR "maxilla"[All Fields] OR "maxillae"[All Fields] OR "maxillas"[All Fields]) OR ("mandible"[MeSH Terms] OR "mandible"[All Fields] OR "mandibles"[All Fields] OR "mandible s"[All Fields]) OR "jaw"[All Fields]) AND ("growth hormone"[MeSH Terms] OR ("growth"[All Fields] AND "hormone"[All Fields]) OR "growth hormone"[All Fields] OR ("growth hormone"[MeSH Terms] OR ("growth"[All Fields] AND "hormone"[All Fields]) OR "growth hormone"[All Fields] OR ("growth"[All Fields] AND "hormones"[All Fields]) OR "growth hormones"[All Fields])) |
| MEDLINE via Ovid  (n=827) | 1 adolescent development/ or childhood development/ or pediatrics/ or exp Congenital Disorders/ or child characteristics/ or child abuse/ or exp child welfare/ or chronically ill children/ or child neglect/ or child psychiatry/ or child psychopathology/ or exp child care/ or (pediatric* or paediatric* or child* or elementary school* or schoolchild* or boy or boys or girl* or middle school* or pubescen* or juvenile* or teen* or youth* or high school* or adolesc* or prepubesc* or pre pubesc*).mp. or (child* or adolesc* or pediat* or paediat*).mp. [mp=title, book title, abstract, original title, name of substance word, subject heading word, floating sub-heading word, keyword heading word, organism supplementary concept word, protocol supplementary concept word, rare disease supplementary concept word, unique identifier, synonyms, population supplementary concept word, anatomy supplementary concept word] (5083650)  2 exp Growth Hormone/ (57136)  3 (Hormon* Supplementation* or Growth Hormone* or Growth Therap* or Hormone Therap*).mp. [mp=title, book title, abstract, original title, name of substance word, subject heading word, floating sub-heading word, keyword heading word, organism supplementary concept word, protocol supplementary concept word, rare disease supplementary concept word, unique identifier, synonyms, population supplementary concept word, anatomy supplementary concept word] (94949)  4 (Craniofacial* or Face* or Facial bone* or Facial phenotyping or maxilla or mandible or jaw*).mp. [mp=title, book title, abstract, original title, name of substance word, subject heading word, floating sub-heading word, keyword heading word, organism supplementary concept word, protocol supplementary concept word, rare disease supplementary concept word, unique identifier, synonyms, population supplementary concept word, anatomy supplementary concept word] (599713)  5 exp Skull/ (214395)  6 exp Mandible/ (61409)  7 exp Maxilla/ (33930)  8 2 or 3 (94949)  9 4 or 5 or 6 or 7 (709214)  10 1 and 8 and 9 (827) |
| EMBASE  via Ovid  (n=1800) | **1**  juvenile/ or exp adolescent/ or exp child/ or exp postnatal development/ or (pediatric* or paediatric* or child* or elementary school* or schoolchild* or toddler* or boy or boys or girl* or middle school* or pubescen* or juvenile* or teen* or youth* or high school* or adolesc* or prepubesc* or pre pubesc*).mp. or (child* or adolesc* or pediat* or paediat*).mp. [mp=title, abstract, heading word, drug trade name, original title, device manufacturer, drug manufacturer, device trade name, keyword heading word, floating subheading word, candidate term word] (4875134)  **2**  exp growth hormone/ (66011)  **3**  (Hormon* Supplementation* or Growth Hormone* or Growth Therap* or Hormone Therap*).mp. [mp=title, abstract, heading word, drug trade name, original title, device manufacturer, drug manufacturer, device trade name, keyword heading word, floating subheading word, candidate term word] (153045)  **4**  exp craniofacial development/ (4296)  **5**  exp skull/ (245198)  **6**  (Craniofacial* or Face* or Facial bone* or Facial phenotyping).mp. [mp=title, abstract, heading word, drug trade name, original title, device manufacturer, drug manufacturer, device trade name, keyword heading word, floating subheading word, candidate term word] (643670)  **7**  exp maxilla/ (38242)  **8**  exp mandible/ (54666)  **9**  (maxilla or mandible or jaw*).mp. [mp=title, abstract, heading word, drug trade name, original title, device manufacturer, drug manufacturer, device trade name, keyword heading word, floating subheading word, candidate term word] (197715)  **10**  2 or 3 (153045)  **11**  4 or 5 or 6 or 7 or 8 or 9 (935448)  **12**  1 and 10 and 11 (1800) |
| WEB OF SCIENCE  (n=1,031) | #1: TOPIC: (pediatric* or paediatric* or child* or newborn* or congenital* or infan* or baby or babies or neonat* or "pre term" or preterm* or "premature birth" or NICU or preschool* or "pre school*" or kindergarten* or "elementary school*" or "nursery school*" or schoolchild* or toddler* or boy or boys or girl* or "middle school*" or pubescen* or juvenile* or teen* or youth* or "high school*" or adolesc* or prepubesc* or pre pubesc*)  Indexes=SCI-EXPANDED, SSCI, A&HCI, CPCI-S, CPCI-SSH, BKCI-S, BKCI-SSH, ESCI, CCR-EXPANDED, IC Timespan=All years  #2: TOPIC: ("Hormon* Supplementation*" or "Growth Hormone*" or "Growth Therap*" or "Hormone Therap*")  Indexes=SCI-EXPANDED, SSCI, A&HCI, CPCI-S, CPCI-SSH, ESCI Timespan=All years  #3: TOPIC: (Craniofacial* or Face* or "Facial bone*" or "Facial phenotyping" or maxilla or mandible or jaw*)  Indexes=SCI-EXPANDED, SSCI, A&HCI, CPCI-S, CPCI-SSH, ESCI Timespan=All years  Final search: #3 AND #2 AND #1 |
| LILACS  (n=313) | ((child*) OR (pediatric*) OR (paediatric*) OR (elementary school*) OR (schoolchild*) OR (boy*) OR (girl*) OR (middle school*) OR (pubescen*) OR (juvenile*) OR (teen*) OR (youth*) OR (adolesc*) OR (pre-pubesc*) OR (prepubesc*)) AND ((Hormone Supplementation*) or (Growth Hormone*) or (Growth Therap*) or (Hormone Therap*)) AND ((Craniofacial*) or (Face*) or (Facial bone*) or (Facial phenotyping) or (maxilla) or (mandible) or (jaw*)) |
| COCHRANE  (N= 388) | (child*) OR (pediatric*) OR (paediatric*) OR (elementary schooler) OR (schoolchild*) OR (boy*) OR (girl*) OR (middle schooler) OR (pubescen*) OR (juvenile*) OR (teen*) OR (youth*) OR (adolesc*) OR (pre-pubesc*) OR (prepubesc*) in Title Abstract Keyword AND (Hormone Supplementation) or (Growth Hormone) or (Growth Hormones) or (Growth Therapy) or (Growth Therapies) or (Hormone Therapy) or (Hormone Therapies) in Title Abstract Keyword AND (Craniofacial*) or (Face*) or (Facial bone) or (Facial bones) or (Facial phenotyping) or (maxilla) or (mandible) or (jaw*) in Title Abstract Keyword - (Word variations have been searched) |
| PROQUEST  (n=128) | noft((child*) OR (pediatric*) OR (paediatric*) OR (elementary schooler) OR (schoolchild*) OR (boy*) OR (girl*) OR (middle schooler) OR (pubescen*) OR (juvenile*) OR (teen*) OR (youth*) OR (adolesc*) OR (pre-pubesc*) OR (prepubesc*)) AND noft((Hormone Supplementation) or (Growth Hormone) or (Growth Hormones) or (Growth Therapy) or (Growth Therapies) or (Hormone Therapy) or (Hormone Therapies)) AND noft((Craniofacial*) or (Face*) or (Facial bone) or (Facial bones) or (Facial phenotyping) or (maxilla) or (mandible) or (jaw*)) |
